# The Impact of Intracoronary Imaging on PCI Outcomes in Cases Utilising Rotational Atherectomy: An Analysis of 8,417 Rotational Atherectomy Cases from the British Cardiovascular Intervention Society Database

**DOI:** 10.1155/2022/5879187

**Published:** 2022-03-15

**Authors:** Majd B. Protty, Sean Gallagher, Andrew S. P. Sharp, Vasim Farooq, Mohaned Egred, Peter O'Kane, Peter Ludman, Mamas A Mamas, Tim Kinnaird

**Affiliations:** ^1^Department of Cardiology, University Hospital of Wales, Cardiff, UK; ^2^Systems Immunity University Research Institute, Cardiff University, Cardiff, UK; ^3^University of Exeter, Exeter, Devon, UK; ^4^Cardiothoracic Department, Freeman Hospital, Newcastle-Upon-Tyne, UK; ^5^Department of Cardiology, Bournemouth Hospital, Bournemouth, UK; ^6^Institute of Cardiovascular Sciences, University of Birmingham, Birmingham, UK; ^7^Keele Cardiovascular Research Group, Institute of Applied Clinical Sciences, University of Keele, Stoke-on-Trent, UK

## Abstract

**Introduction:**

There is increasing evidence supporting the use of intracoronary imaging to optimize the outcomes of percutaneous coronary intervention (PCI). However, there are no studies examining the impact of imaging on PCI outcomes in cases utilising rotational atherectomy (RA-PCI). Our study examines the determinants and outcomes of using intracoronary imaging in RA-PCI cases including 12-month mortality.

**Methods:**

Using the British Cardiac Intervention Society database, data were analysed on all RA-PCI procedures in the UK between 2007 and 2014. Descriptive statistics and multivariate logistic regressions were used to examine baseline, procedural, and outcome associations with intravascular imaging.

**Results:**

Intracoronary imaging was used in 1,279 out of 8,417 RA-PCI cases (15.2%). Baseline covariates associated with significantly more imaging use were number of stents used, smoking history, previous CABG, pressure wire use, proximal LAD disease, laser use, glycoprotein inhibitor use, cutting balloons, number of restenosis attempted, off-site surgery, and unprotected left main stem (uLMS) PCI. Adjusted rates of in-hospital major adverse cardiac/cerebrovascular events (IH-MACCE), its individual components (death, peri-procedural MI, stroke, and major bleed), or 12-month mortality were not significantly altered by the use of imaging in RA-PCI. However, subgroup analysis demonstrated a signal towards reduction in 12-month mortality in uLMS RA-PCI cases utilising intracoronary imaging (OR 0.67, 95% CI 0.44–1.03).

**Conclusions:**

Intracoronary imaging use during RA-PCI is associated with higher risk of baseline and procedural characteristics. There were no differences observed in IH-MACCE or 12-month mortality with intracoronary imaging in RA-PCI.

## 1. Introduction

The use of intracoronary imaging has been increasingly recognized as an important tool to optimize outcomes following percutaneous coronary intervention (PCI) [1, 2]. These include the use of intravascular ultrasound (IVUS) or optical coherence tomography (OCT). Both of these modalities have been shown to be particularly useful in characterising calcified atheromas by providing precise lesion assessment assessing the axial, circumferential, and longitudinal distribution of calcium [3, 4]. This in turn allows operators to decide on the choice of tools and calcium modification strategies to employ [4].

Rotational atherectomy (RA-PCI) continues to form part of the calcium modification algorithms used to treat coronary lesions which are heavily calcified [5, 6]. Increasingly, the use of intracoronary imaging as part of RA-PCI has been shown to improve the safety profile of the procedure and achieve optimal stent expansion [7, 8], with expert consensus agreeing that intracoronary imaging maximizes efficacy for RA-PCI without sacrificing safety [9]. However, there is very limited evidence on how intracoronary imaging influences morbidity and mortality beyond procedural outcomes in patients undergoing RA-PCI [7, 10]. Therefore, in this study, we aimed to investigate the “real-world” patterns, predictors, and outcomes of intracoronary imaging use in RA-PCI using a national PCI database.

## 2. Methods

### 2.1. Study Design, Setting, and Participants

We retrospectively analysed national data from all patients undergoing RA-PCI in the United Kingdom between January 2007 and December 2014, as described previously [11]. During the study period, a total of 8,417 cases underwent RA-PCI and were eligible for inclusion (see Supplementary Figure S1). The study design was approved by the review board of the National Institute of Clinical Outcomes Research (NICOR) and data release approved by the Healthcare Quality Improvement Partnership (HQIP).

### 2.2. Setting, Data Source, and Study Size

Data on RA-PCI practice in the United Kingdom were obtained from the National PCI Audit dataset that records this information prospectively and publishes this information in the public domain as part of the national transparency agenda [12]. The data collection process is overseen by NICOR (http://www.ucl.ac.uk/nicor/) with high levels of case ascertainment. The database contains 121 clinical, procedural, and outcome variables, and in 2014, 98.6% of all PCI procedures performed in the National Health Service hospitals in the United Kingdom (http://www.bcis.org.uk/) were recorded on the database, with approximately 100,000 new records currently added each year. The accuracy of and quality of the BCIS dataset has previously been ascertained [13].

Entry of all PCI procedures by UK interventional operators is mandated as part of professional revalidation. The participants of the database are tracked by the Medical Research Information Services for subsequent mortality using the patients' National Health Service (NHS) number (a unique identifier for any person registered within the NHS in England and Wales).

### 2.3. Study Definitions

We analysed all recorded RA-PCI procedures that were undertaken in the United Kingdom between January 1st, 2007, and December 31st, 2014. Study definitions were used as in the National PCI database. Specifically, preprocedural renal failure is defined as any one of the following: creatinine >200 *µ*mol/l, renal transplant history, or dialysis. Pre- or post-PCI disease severity was defined as a stenosis ≥50% in the case of the left main artery. Intravascular imaging is a combination of intravascular ultrasound (IVUS) and optical coherence tomography (OCT). An access site complication was defined as either a false aneurysm, haemorrhage (without haematoma), haemorrhage with delayed hospital discharge, retroperitoneal haematoma, arterial dissection, or any access site complication requiring surgical repair. The clinical outcomes examined were in-hospital mortality, in-hospital MACCE (defined as a combination of death, peri-procedural ischaemic stroke, or peri-procedural myocardial infarction after PCI), in-hospital major bleeding (defined as either gastrointestinal bleed, intracerebral bleed, retroperitoneal haematoma, blood or platelet transfusion, access site haemorrhage, or an access site complication requiring surgery), in-hospital emergency cardiac surgery, tamponade, side branch loss, dissection, perforation, heart block, slow flow, peri-procedural shock, and access site complications.

### 2.4. Data Analyses

The study flow is illustrated in Supplementary Figure S1. Statistical analysis was performed using the *R* coding environment (Open Source). Multiple imputations were carried out using the *mice* package to reduce the potential bias from missing data (Supplementary Table S1), assuming missingness at random mechanisms. We used chained equations to impute the data for all variables with missing information and generated 5 datasets to be used in the analyses. We examined the baseline and procedural characteristics of participants by intracoronary imaging status. We explored crude baseline comorbidities using a Chi-squared test for categorical variables and the Wilcoxon–Mann–Whitney test for continuous variables.

A multiple logistic regression model was developed to identify variables associated with intra-coronary imaging in RA-PCI. The potential predictor variables in the model included age, gender, weight, number of disease vessels pre-PCI, acute coronary syndrome (ACS) status and type, CCS score, NYHA score, renal disease (CKD: chronic kidney disease), clopidogrel use, prasugrel use, ticagrelor use, warfarin use, stroke, diabetes, ejection fraction (EF) <30%, hypertension, off-site surgery, peripheral vascular disease (PVD), smoking history, valve disease, previous myocardial infarction (MI), previous coronary artery bypass grafting (CABG), previous PCI, ventilated preprocedure, Q-wave on ECG, number of vessels attempted, number of lesions attempted, number of chronic total occlusions (CTO) attempted, number of restenosis attempted, number of stents used, unprotected left main stem (uLMS) disease, proximal left anterior descending (LAD) disease, glycoprotein inhibitor use, pressure wire use, laser use, cutting balloon use, aspiration catheter use, emboli protection device use, intra-aortic balloon pump use, and femoral access.

To examine the influence of intracoronary imaging on RA-PCI outcomes, we built on and included the previously described baseline model to investigate the independent odds of in-hospital major adverse cardiac/cerebrovascular events (IH-MACCE), in-hospital death, 12-month mortality, peri-procedural MI, postprocedural stroke, transfusion, tamponade, emergency CABG, acute kidney injury, in-hospital major bleed, side branch loss, dissection, perforation, heart block, DC cardioversion, dissection, slow flow, shock induction, access site complication, arterial haemorrhage, and gastrointestinal (GI) bleed. A sensitivity analysis was also carried out to study the outcomes using a propensity-score matched analysis. This was performed using the MatchIt *R* package on a 1 : 1 matching basis using nearest neighbour.

Finally, a subgroup analysis of 12-month mortality for high risk groups, defined as ACS, CKD, CTO, diabetes, stent diameter <2.75 mm, stent length >60 mm, and uLMS, was carried out using similar methodology to above, accounting for interaction between these variables and 12-month mortality.

## 3. Results

### 3.1. Utility of Intracoronary Imaging in RA-PCI and Baseline Demographics of the Study Population

**Figure 1:**
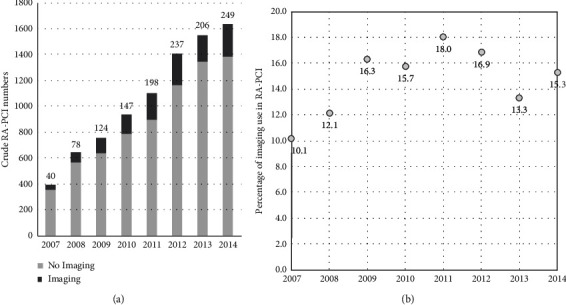
Trends in intravascular imaging use in RA-PCI performed in England and Wales, 2007–2014. (a) Crude numbers of imaging (dark grey bars) and nonimaging RA-PCI cases (light grey bars); (b) percentage of RA-PCI cases utilising imaging as a proportion of all RA-PCI demonstrates an initial rise followed by a plateau (*p* for trend <0.001).

Crude numbers per year of RA-PCI increased during the initial study period from 395 in 2007 to 1632 in 2014 with a corresponding increase in the use of intracoronary imaging during RA-PCI from 40 in 2007 to 249 in 2014 (Figure 1(a)). The proportion of intracoronary imaging use in RA-PCI increased from 10.1% (2004) to 16.3% (2009), followed by a plateau averaging 15.2% utility (Figure 1(b)). The baseline characteristics of RA-PCI patients with and without intracoronary imaging use are presented in Table 1. Intracoronary imaging use in RA-PCI was associated with lower age, high CCS and NYHA scores, higher number of diseased vessels pre-PCI, history of stroke, ACS, NSTEMI, off-site surgery, and smoking history (*p* < 0.05, unadjusted).

### 3.2. Procedural Variables during RA-PCI by Intracoronary Imaging Use

Procedural variables for RA-PCI patients with and without intracoronary imaging use are presented in Table 2. RA-PCI cases when imaging was used were associated with a higher number of vessels, lesions and restenosis attempted, presence of uLMS or proximal LAD disease, use of glycoprotein inhibitors, pressure wires, laser, cutting balloons, intra-aortic balloon pumps, and a higher number of stents used (*p* < 0.05, unadjusted). Femoral access was less likely to be used in RA-PCI cases (*p* < 0.05, unadjusted).

### 3.3. Predictors of Intracoronary Imaging Use during RA-PCI in England and Wales, 2007–2014

**Figure 2:**
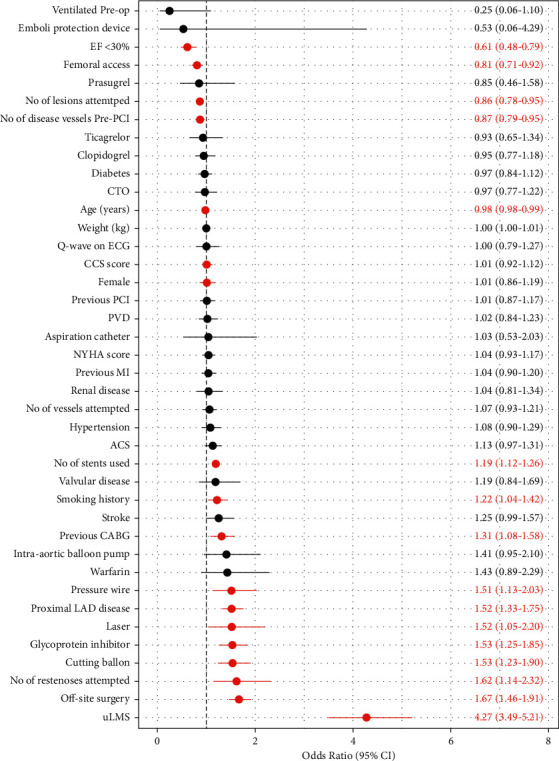
Multivariate logistic regression for intracoronary imaging use by baseline comorbidity in patients undergoing RA-PCI in England and Wales, 2007−2014. CI: confidence interval.

After adjusting for baseline comorbidities using a multivariate analysis, several factors remained significantly associated with intracoronary use (Figure 2). Factors associated with higher intracoronary imaging use in RA-PCI number of stents used, smoking history, previous CABG, use of pressure wire, laser, glycoprotein inhibitor, cutting balloon, presence of proximal LAD or uLMS disease, higher number of restenosis attempted, and off-site surgery (*p* < 0.05). Variables with a lower likelihood of intracoronary imaging use in RA-PCI were EF <30%, femoral access, number of lesions attempted, and number of diseased vessels pre-PCI (*p* < 0.05).

### 3.4. Clinical Outcomes of RA-PCI by Intracoronary Imaging Use

The unadjusted incidence of procedural complications and outcomes associated with intracoronary imaging use in RA-PCI is shown in Table 3. Complications crudely associated with intracoronary imaging use were dissection, shock induction, access site complications, higher number of lesions successful, lower number of residual diseased vessels post-PCI, and longer length of hospital stay (*p* < 0.05, unadjusted).

**Figure 3:**
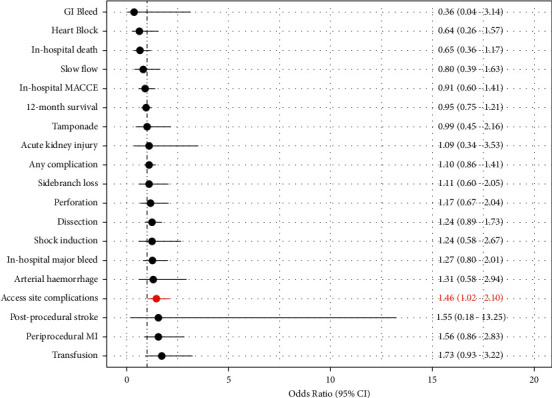
Adjusted outcomes by intracoronary imaging use in patients undergoing RA-PCI in England and Wales, 2007−2014. CI: confidence interval; MACCE: major adverse cardiac and cerebrovascular events.

Multivariate logistic modelling of outcomes was used to adjust outcomes for baseline comorbidities (Figure 3). This showed that intracoronary imaging use during RA-PCI was significantly (*p* < 0.05) associated with higher likelihood of access site complications (OR 1.46, 95% CI 1.02–2.10) with no significant increase in in-hospital MACCE or 12-month survival. Sensitivity analysis using propensity-matched cohorts also confirmed no difference in in-hospital MACCE or 12-month survival in cases utilising intravascular imaging (Supplementary Table S2).

**Figure 4:**
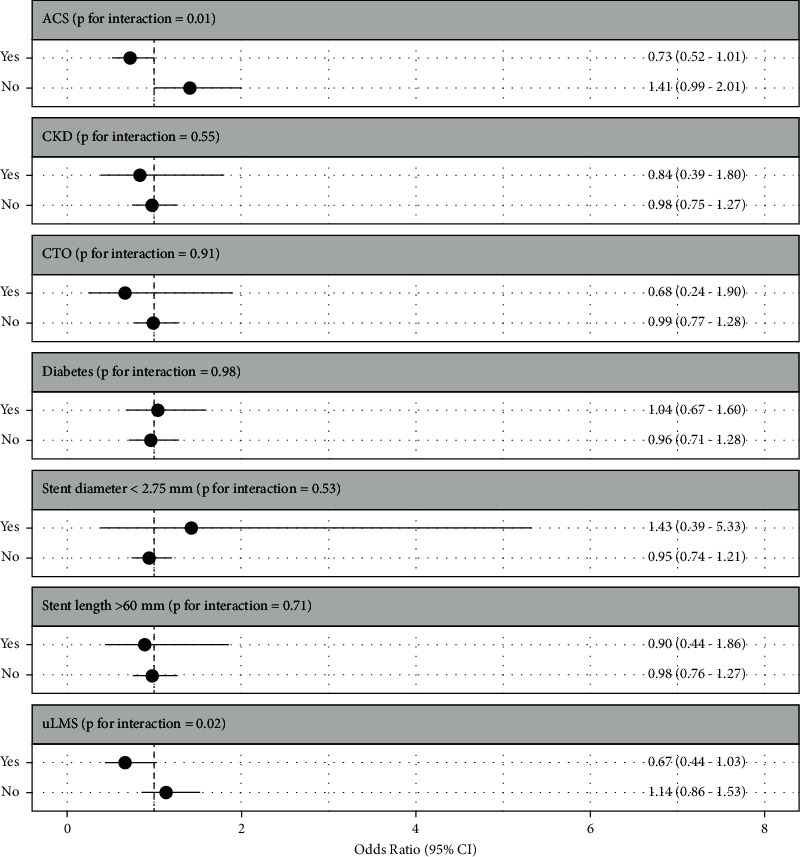
Subgroup analysis of adjusted 12-month mortality by intracoronary imaging use in RA-PCI in England and Wales, 2007−2014. CI: confidence interval; MACCE: major adverse cardiac and cerebrovascular events.

A subgroup analysis of 12-month mortality in RA-PCI cases demonstrated no difference in 12-month mortality for the examined risk groups; however, it did suggest a signal of benefit in uLMS cases (OR 0.67, 95% CI 0.44–1.03) (Figure 4).

## 4. Discussion

This study utilises a large national cohort of RA-PCI to investigate patterns of use, predictors, and outcomes of intracoronary imaging over an eight-year follow-up period (2007–2014). We demonstrate that the average rate of intracoronary imaging use in RA-PCI during the study period was 15.2%. Following adjustment for baseline and procedural variables, intracoronary imaging use in RA-PCI did not alter in-hospital MACCE or 12-month mortality.

The rate of intracoronary imaging use in RA-PCI showed an initial increase in 2007–2009, followed by a plateau. This likely represents the natural history of operators gaining more expertise and confidence in using intracoronary imaging in the management of calcific disease [5]. Nevertheless, the overall uptake of intracoronary imaging in RA-PCI remained low at an average of 15.2%. This is consistent with the reported utility of intracoronary imaging in “real-world” UK practice of <15% in all comer PCI, despite mounting evidence of the benefit of intracoronary imaging in improving MACCE outcomes [14–16]. There are multiple reasons for why this could be the case, including cost and availability. However, focusing on RA-PCI, one of the main limitations to using intracoronary imaging is whether the lesions are balloon-crossable or uncrossable, the latter being a main indication for RA-PCI [17]. In fact, studies have shown “IVUS-crossability” as a possible tool for further risk stratification of RA-PCI cases with reduced complications [18]. Nevertheless, our study has shown no impact of intracoronary imaging on in-hospital MACCE or 12-month mortality, which may suggest that the value of intracoronary imaging in RA-PCI beyond lesion characterisation is limited. This may be due to the nature of the calcific disease mandating the use of RA-PCI which may be a stronger determinant of outcome in this patient group and which requires more than RA-PCI to modify prognosis [19, 20]. Supporting this are studies on the use of IVUS in patients undergoing orbital atherectomy PCI, which demonstrated no difference in 3-year MACCE outcomes compared with angiography guidance only [21]. Reassuringly, however, adjusted rates of procedural complications were also unchanged with the use of intracoronary imaging, further emphasizing its safety [22–24]. The only exception to this is access site complications which were higher in RA-PCI utilising imaging (OR 1.46, 95% CI 1.02–2.10), as seen in Figure 3. This may be related to the unmeasured confounder of sheath/burr size which is not recorded in the BCIS database and therefore unadjusted for. It is possible that RA-PCI operators utilising imaging opt for larger sheath sizes upfront to accommodate equipment and larger burrs, which could explain the higher access site complications observed in Figure 3.

Adjusted modelling of baseline and procedural variables highlights the association of intracoronary imaging use with other calcium modifying strategies (cutting balloons and laser), in PCI to prognostic vessels (proximal LAD and uLMS) and in the presence of high risk factors such as previous CABG, smoking history, and off-site surgery. These covariates are indicative of relatively higher complexity and risk, forming the basis for using imaging. However, we noted lower odds of utility in older patients, EF <30%, and those with fewer number of diseased vessels pre-PCI and lesions attempted. This may suggest an element of case selection bias whereby older patients with impaired left ventricles and more limited disease distribution are not selected to undergo intracoronary imaging during RA-PCI. The other explanation is the presence of unmeasured clinical factors such as frailty and/or poor patient tolerance of the RA-PCI procedure which could influence the operator's decision [25]. In any case, whether this apparent bias interferes with outcomes is not known.

Given the heterogenous nature of patients undergoing RA-PCI, we carried out subgroup analyses of 12-month mortality on patients who are known to be more complex (CTO, CKD, uLMS, ACS, and diabetes) or in whom intracoronary imaging may affect the optimal sizing of treated segments (Stent length >60 mm or diameter <2.75 mm). There was no difference in 12-month mortality in these RA-PCI groups. The signal of benefit in the uLMS RA-PCI subgroup is consistent with the evidence of benefit in intracoronary imaging use shown previously by us and by other groups [1, 26]. This is thought to be related to the large diameter stents whose deployment, expansion, and apposition need to be as perfect as possible to avoid complications such as stent thrombosis [1, 26]. Nevertheless, it is likely that intravascular imaging is still very useful with calcified coronary anatomy to enable identification of patterns of calcium (circumferential vs nodular, superficial vs deep) which may guide selection of the most appropriate calcium modification technique (e.g., rotational atherectomy, lithotripsy, or cutting balloons) [4, 5].

The major strength of this study is that the BCIS National PCI data set includes >98% of all PCI procedures performed in the United Kingdom, which, therefore, reflects a national, real-world experience that includes high-risk patients encountered in daily interventional practice (who are often excluded from randomized controlled trials). There are several limitations to this study. Firstly, the BCIS database does not record the technical elements surrounding RA-PCI such as burr size, rotational speeds, or the achievement of calcium fracture. Secondly, the BCIS database does not capture intracoronary imaging data, meaning that we cannot provide details of minimal luminal and stent area and correlate these with outcomes. Furthermore, the BCIS database does not allow us to distinguish between upfront imaging use or use later in the procedure. Moreover, it was not possible to look at OCT-only outcomes in this 2007–2014 dataset due to the relatively smaller numbers of OCT compared with IVUS in this time period, hindering us from carrying out more detailed analysis of individual imaging modalities. Finally, as with any observational data, whilst the statistical adjustment aims to correct for baseline differences and complexity, confounders may remain which could influence the study findings. Consequently, conclusions need to be interpreted in the context of the observational nature of these findings.

## 5. Conclusions

Intracoronary imaging is utilised in 15.2% of RA-PCI, and its use is associated with higher risk baseline and procedural characteristics. Whilst there were no differences observed in IH-MACCE or 12-month mortality with intracoronary imaging in RA-PCI, subgroup analysis suggested a signal towards lower odds of 12-month mortality in uLMS RA-PCI cases.

## Figures and Tables

**Table 1:** Baseline participant characteristics by intracoronary imaging use in patients undergoing RA-PCI in England and Wales, 2007−2014.

Variable	All (*n* = 8417)	No imaging (*n* = 7138)	Imaging (*n* = 1279)	*p* value
Age (years), ±SD	73.1 ± 9.3	73.2 ± 9.3	72.6 ± 9.3	0.03
Female gender, no. (%)	2356 (28)	2024 (28.4)	332 (26)	0.09
Weight (kg), ±SD	80.8 ± 17.3	80.6 ± 17.1	81.7 ± 18.4	0.21
CCS score, ±SD	2.5 ± 1	2.5 ± 1	2.6 ± 1.1	<0.01
NYHA score, ±SD	2.1 ± 0.9	2.1 ± 0.9	2.2 ± 0.9	0.01
No. of diseased vessels pre-PCI, ±SD	1.7 ± 0.9	1.7 ± 0.9	1.8 ± 0.9	<0.01
Renal disease, no. (%)	562 (6.9)	469 (6.8)	93 (7.5)	0.40
Clopidogrel, no. (%)	6890 (84.5)	5857 (84.6)	1033 (84.3)	0.84
Prasugrel, no. (%)	87 (1.1)	72 (1)	15 (1.2)	0.62
Ticagrelor, no. (%)	344 (4.2)	290 (4.2)	54 (4.4)	0.81
Stroke, no. (%)	622 (8.8)	499 (8.4)	123 (11)	0.01
Diabetes, no. (%)	2529 (30.7)	2138 (30.6)	391 (31.2)	0.69
Ejection fraction <30%, no. (%)	527 (9.7)	447 (10)	80 (8.4)	0.15
Hypertension, no. (%)	6015 (84.8)	5057 (84.7)	958 (85.6)	0.47
ACS, no. (%)	3265 (38.8)	2718 (38.1)	547 (42.8)	<0.01
STEMI, no. (%)	112 (1.4)	101 (1.5)	11 (0.9)	0.13
NSTEMI, no. (%)	3067 (38.1)	2554 (37.3)	513 (42.3)	<0.01
Off-site surgery, no. (%)	2783 (34.9)	2251 (32.9)	532 (47.1)	<0.01
PVD, no. (%)	985 (13.9)	812 (13.6)	173 (15.5)	0.10
Smoking history, no. (%)	4804 (62.3)	3993 (61.4)	811 (67.4)	<0.01
Valve disease, no. (%)	340 (4.8)	280 (4.7)	60 (5.4)	0.36
Warfarin, no. (%)	131 (1.6)	105 (1.5)	26 (2.1)	0.15
Previous MI, no. (%)	3260 (41.6)	2769 (41.1)	491 (44.6)	0.03
Previous CABG, no. (%)	1270 (15.3)	1079 (15.3)	191 (15.1)	0.89
Previous PCI, no. (%)	2727 (33)	2309 (32.9)	418 (33.3)	0.80
Ventilated preprocedure, no. (%)	39 (0.5)	37 (0.6)	2 (0.2)	0.17
Q-wave on ECG, no. (%)	839 (11.2)	712 (11.1)	127 (11.9)	0.47
*Year*				
2007	395 (4.7)	355 (5)	40 (3.1)	<0.01
2008	644 (7.7)	566 (7.9)	78 (6.1)	0.03
2009	759 (9)	635 (8.9)	124 (9.7)	0.39
2010	935 (11.1)	788 (11)	147 (11.5)	0.63
2011	1098 (13)	900 (12.6)	198 (15.5)	0.01
2012	1405 (16.7)	1168 (16.4)	237 (18.5)	0.07
2013	1549 (18.4)	1343 (18.8)	206 (16.1)	0.02
2014	1632 (19.4)	1383 (19.4)	249 (19.5)	0.97

SD: standard deviation; PVD: peripheral vascular disease; MI: myocardial infarction; PCI: percutaneous coronary intervention; CABG: coronary artery bypass graft; ACS: acute coronary syndrome; NSTEMI: non-ST elevation MI; STEMI: ST-elevation MI.

**Table 2:** Procedural variables by intracoronary imaging use in patients undergoing RA-PCI in England and Wales, 2007−2014.

Variable	All (*n* = 8417)	No imaging (*n* = 7138)	Imaging (*n* = 1279)	*p* value
No. of vessels attempted, ±SD	1.4 ± 0.6	1.3 ± 0.6	1.6 ± 0.8	<0.01
No. of lesions attempted, ±SD	1.6 ± 0.8	1.6 ± 0.8	1.8 ± 0.9	<0.01
No. of chronic total occlusions attempted, ±SD	0.1 ± 0.4	0.1 ± 0.4	0.1 ± 0.4	0.78
CTO, no. (%)	751 (9.3)	643 (9.4)	108 (9)	0.70
No. of restenosis attempted, no. (%)	0 ± 0.2	0 ± 0.2	0 ± 0.2	<0.01
LMS disease pre-PCI, no. (%)	925 (11)	590 (8.3)	335 (26.2)	<0.01
Proximal LAD disease pre-PCI, no. (%)	3533 (42.1)	2836 (39.8)	697 (54.6)	<0.01
Glycoprotein inhibitor, no. (%)	865 (10.8)	682 (10)	183 (15.4)	<0.01
Pressure wire use, no. (%)	311 (3.7)	242 (3.4)	69 (5.4)	<0.01
Laser, no. (%)	199 (2.4)	156 (2.2)	43 (3.4)	0.01
Cutting balloon use, no. (%)	638 (7.6)	512 (7.2)	126 (9.9)	<0.01
Aspiration catheter, no. (%)	63 (0.8)	51 (0.7)	12 (1)	0.35
Emboli protection device, no. (%)	11 (0.1)	10 (0.1)	1 (0.1)	1.00
Intra-aortic balloon pump use, no. (%)	183 (2.2)	139 (2)	44 (3.6)	<0.01
Femoral access, no. (%)	5178 (62.1)	4451 (63)	727 (57.3)	<0.01
No. of stents used, ±SD	2 ± 1.2	2 ± 1.2	2.3 ± 1.3	<0.01

SD: standard deviation; CTO: chronic total occlusion.

**Table 3:** Crude outcomes by intracoronary imaging use in patients undergoing RA-PCI in England and Wales, 2007−2014.

Variable	All (*n* = 8417)	No imaging (*n* = 7138)	Imaging (*n* = 1279)	*p* value
Transfusion, no. (%)	68 (0.8)	53 (0.7)	15 (1.2)	0.09
Postprocedural stroke, no. (%)	8 (0.1)	7 (0.1)	1 (0.1)	1.00
Emergency CABG, no. (%)	6 (0.1)	6 (0.1)	0 (0)	-
GI bleed, no. (%)	9 (0.1)	8 (0.1)	1 (0.1)	1.00
Periprocedural MI, no. (%)	77 (0.9)	62 (0.9)	15 (1.2)	0.40
Acute kidney injury, no. (%)	18 (0.2)	14 (0.2)	4 (0.3)	0.69
Tamponade, no. (%)	51 (0.6)	42 (0.6)	9 (0.7)	0.82
In-hospital death, no. (%)	113 (1.3)	97 (1.4)	16 (1.3)	0.89
In-hospital major bleed, no. (%)	139 (1.7)	113 (1.6)	26 (2)	0.36
In-hospital MACCE, no. (%)	192 (2.3)	162 (2.3)	30 (2.3)	1.00
Dissection, no. (%)	257 (3.1)	204 (2.9)	53 (4.1)	0.03
Perforation, no. (%)	91 (1.1)	73 (1)	18 (1.4)	0.25
Heart block, no. (%)	54 (0.6)	48 (0.7)	6 (0.5)	0.55
Slow flow, no. (%)	67 (0.8)	57 (0.8)	10 (0.8)	1.00
Sidebranch loss, no. (%)	74 (0.9)	60 (0.8)	14 (1.1)	0.36
Shock induction, no. (%)	43 (0.5)	32 (0.4)	11 (0.9)	0.03
Any complication, no. (%)	519 (6.2)	423 (5.9)	96 (7.5)	0.03
Access site complications, no. (%)	206 (2.5)	162 (2.3)	44 (3.6)	0.01
Arterial haemorrhage, no. (%)	44 (0.5)	36 (0.5)	8 (0.6)	0.79
12-month survival, no. (%)	689 (8.2)	582 (8.2)	107 (8.4)	0.86
No. of lesions successful, ±SD	1.6 ± 0.8	1.5 ± 0.8	1.7 ± 0.9	<0.01
Residual diseased vessels post-PCI, ±SD	0.5 ± 0.8	0.6 ± 0.8	0.5 ± 0.8	0.04
Length of hospital stay (days), ±SD	3.1 ± 7.1	3 ± 7.1	3.3 ± 6.9	0.04

## Data Availability

The BCIS PCI data used to support the findings of this study are restricted by BCIS/NICOR in order to protect patient privacy. Anonymised data are available from BCIS/NICOR for researchers who meet the criteria for accessing confidential data.

## Supplementary Materials

Supplementary Materials Supplementary Figure S1: consort flow diagram for study population. Supplementary Table S1: percentage of missing data in baseline, procedural, and outcome variables. Supplementary Table S2: propensity-matched analysis of rotational atherectomy cases with or without intracoronary imaging (1:1 matching) demonstrates no differences in outcomes between the two groups. .
