# Supplementary material for: The Impact of Intracoronary Imaging on PCI Outcomes in Cases Utilising Rotational Atherectomy: An Analysis of 8,417 Rotational Atherectomy Cases from the British Cardiovascular Intervention Society Database
Source: J Interv Cardiol. 2022 Mar 15;2022:5879187. doi: 10.1155/2022/5879187 (PMC8941577; doi:10.1155/2022/5879187)
Supplement: Supplementary Materials — Supplementary Figure S1: consort flow diagram for study population. Supplementary Table S1: percentage of missing data in baseline, procedural, and outcome variables. Supplementary Table S2: propensity-matched analysis of rotational atherectomy cases with or without intracoronary imaging (1:1 matching) demonstrates no differences in outcomes between the two groups. . [file 5879187.f1.zip › 5879187.f1/Supp Table S1.docx]

| **Supplementary Table S1.** Percentage of missing data in baseline, procedural and outcome variables | |
| --- | --- |
| **Variable** | **Percent (%) missing** |
| Age | 0.0 |
| CCS Score | 22.4 |
| NYHA Score | 24.2 |
| Weight | 30.9 |
| No of diseased vessels pre-PCI | 8.3 |
| No of vessels attempted | 0.0 |
| No of lesions attempted | 0.0 |
| No of CTO attempted | 3.9 |
| No of restenoses attempted | 4.3 |
| No of stents used | 0.1 |
| No of lesions successful | 0.0 |
| Residual diseased vessels post-PCI | 6.9 |
| Length of hospital stay | 5.7 |
| Intracoronary imaging | 0.0 |
| Female Gender | 0.2 |
| ACS | 0.0 |
| NSTEMI | 4.0 |
| STEMI | 4.0 |
| Previous MI | 12.6 |
| Previous CABG | 1.1 |
| Previous PCI | 1.9 |
| Diabetes | 2.6 |
| EF <30% | 28.3 |
| Smoking history | 8.3 |
| Hypertension | 18.0 |
| Stroke | 18.0 |
| PVD | 18.0 |
| Valve disease | 18.0 |
| Renal disease | 4.4 |
| Ventilated Pre-Op | 9.2 |
| Q-wave on ECG | 10.5 |
| Clopidogrel | 1.7 |
| Prasugrel | 1.7 |
| Ticagrelor | 1.7 |
| Warfarin | 1.7 |
| Off-site surgery | 9.4 |
| uLMS | 0.0 |
| Proximal LAD | 0.0 |
| CTO | 3.9 |
| Glycoprotein inhibitor use | 2.2 |
| Pressure wire | 0.0 |
| Rotational atherectomy | 0.0 |
| Laser | 0.0 |
| Cutting balloons | 0.0 |
| Aspiration catheter | 0.6 |
| Emboli protection device | 0.6 |
| Intra-aortic balloon pump | 2.5 |
| Femoral access | 0.6 |
| Transfusion | 0.0 |
| Postprocedural stroke | 0.0 |
| Emergency CABG | 0.0 |
| GI Bleed | 0.0 |
| Periprocedural MI | 0.0 |
| AKI | 0.0 |
| Tamponade | 0.0 |
| In-hospital death | 0.0 |
| In-hospital major bleed | 0.0 |
| In-hospital MACCE | 0.0 |
| Dissection | 0.0 |
| Perforation | 0.0 |
| Heartblock | 0.0 |
| Slow flow | 0.0 |
| Sidebranch loss | 0.0 |
| Shock induction | 0.0 |
| Any complication | 0.0 |
| All complications | 0.0 |
| Arterial haemorrhage | 0.0 |
| 12-month survival | 0.0 |
