# Supplementary material for: The Impact of Intracoronary Imaging on PCI Outcomes in Cases Utilising Rotational Atherectomy: An Analysis of 8,417 Rotational Atherectomy Cases from the British Cardiovascular Intervention Society Database
Source: J Interv Cardiol. 2022 Mar 15;2022:5879187. doi: 10.1155/2022/5879187 (PMC8941577; doi:10.1155/2022/5879187)
Supplement: Supplementary Materials — Supplementary Figure S1: consort flow diagram for study population. Supplementary Table S1: percentage of missing data in baseline, procedural, and outcome variables. Supplementary Table S2: propensity-matched analysis of rotational atherectomy cases with or without intracoronary imaging (1:1 matching) demonstrates no differences in outcomes between the two groups. . [file 5879187.f1.zip › 5879187.f1/Supp Table S2.docx]

**Supplementary Table S2.** Propensity-matched analysis of rotational atherectomy cases with or without intracoronary imaging (1:1 matching) demonstrates no differences in outcomes between the two groups.

| **Variable** | **No Imaging** | **Imaging** | **p** |
| --- | --- | --- | --- |
| **n** | **1279** | **1279** |  |
| Age, mean (SD) | 72.86 (9.53) | 72.56 (9.32) | 0.42 |
| CCS Score, mean (SD) | 2.67 (0.94) | 2.64 (1.04) | 0.52 |
| NYHA Score, mean (SD) | 2.25 (0.92) | 2.24 (0.92) | 0.61 |
| Weight, mean (SD) | 81.98 (18.13) | 82.27 (18.38) | 0.69 |
| No of diseased vessels pre-PCI, mean (SD) | 1.79 (0.95) | 1.80 (0.93) | 0.95 |
| No of vessels attempted, mean (SD) | 1.55 (0.79) | 1.57 (0.77) | 0.51 |
| No of lesions attempted, mean (SD) | 1.76 (0.96) | 1.77 (0.91) | 0.77 |
| No of CTO attempted, mean (SD) | 0.09 (0.40) | 0.11 (0.40) | 0.32 |
| No of restenoses attempted, mean (SD) | 0.03 (0.20) | 0.04 (0.20) | 0.43 |
| No of stents used, mean (SD) | 2.33 (1.32) | 2.32 (1.30) | 0.92 |
| No of lesions successful, mean (SD) | 1.70 (0.95) | 1.74 (0.91) | 0.24 |
| Residual diseased vessels post-PCI, mean (SD) | 0.52 (0.82) | 0.48 (0.76) | 0.30 |
| Length of hospital stay, mean (SD) | 2.94 (3.91) | 3.24 (6.80) | 0.17 |
| Intracoronary imaging, n (%) | 0 (0.0) | 1279 (100.0) | <0.001 |
| Female Gender, n (%) | 327 (25.6) | 333 (26.0) | 0.82 |
| ACS, n (%) | 535 (41.8) | 547 (42.8) | 0.66 |
| NSTEMI, n (%) | 516 (40.3) | 536 (41.9) | 0.45 |
| STEMI, n (%) | 19 (1.5) | 11 (0.9) | 0.20 |
| Previous MI, n (%) | 571 (44.6) | 553 (43.2) | 0.50 |
| Previous CABG, n (%) | 194 (15.2) | 192 (15.0) | 0.96 |
| Previous PCI, n (%) | 415 (32.4) | 427 (33.4) | 0.64 |
| Diabetes, n (%) | 382 (29.9) | 399 (31.2) | 0.49 |
| EF <30%, n (%) | 99 (7.7) | 95 (7.4) | 0.82 |
| Smoking history, n (%) | 872 (68.2) | 860 (67.2) | 0.64 |
| Hypertension, n (%) | 1093 (85.5) | 1090 (85.2) | 0.91 |
| Stroke, n (%) | 143 (11.2) | 145 (11.3) | 0.95 |
| PVD, n (%) | 203 (15.9) | 196 (15.3) | 0.74 |
| Valve disease, n (%) | 72 (5.6) | 70 (5.5) | 0.93 |
| Renal disease, n (%) | 105 (8.2) | 99 (7.7) | 0.72 |
| Ventilated Pre-Op, n (%) | 5 (0.4) | 3 (0.2) | 0.72 |
| Q-wave on ECG, n (%) | 156 (12.2) | 151 (11.8) | 0.81 |
| Clopidogrel, n (%) | 1069 (83.6) | 1076 (84.1) | 0.75 |
| Prasugrel, n (%) | 18 (1.4) | 16 (1.3) | 0.86 |
| Ticagrelor, n (%) | 60 (4.7) | 56 (4.4) | 0.78 |
| Warfarin, n (%) | 22 (1.7) | 26 (2.0) | 0.66 |
| Off-site surgery, n (%) | 590 (46.1) | 596 (46.6) | 0.84 |
| uLMS, n (%) | 342 (26.7) | 335 (26.2) | 0.79 |
| Proximal LAD, n (%) | 667 (52.2) | 697 (54.5) | 0.25 |
| CTO, n (%) | 101 (7.9) | 113 (8.8) | 0.43 |
| Glycoprotein inhibitor use, n (%) | 190 (14.9) | 198 (15.5) | 0.70 |
| Pressure wire, n (%) | 63 (4.9) | 69 (5.4) | 0.66 |
| Rotational atherectomy, n (%) | 1279 (100.0) | 1279 (100.0) | - |
| Laser, n (%) | 36 (2.8) | 43 (3.4) | 0.49 |
| Cutting balloons, n (%) | 116 (9.1) | 126 (9.9) | 0.54 |
| Aspiration catheter, n (%) | 6 (0.5) | 12 (0.9) | 0.24 |
| Emboli protection device, n (%) | 1 (0.1) | 1 (0.1) | 1.00 |
| Intra-aortic balloon pump, n (%) | 45 (3.5) | 44 (3.4) | 1.00 |
| Femoral access, n (%) | 730 (57.1) | 736 (57.5) | 0.84 |
| Transfusion, n (%) | 17 (1.3) | 15 (1.2) | 0.86 |
| Postprocedural stroke, n (%) | 0 (0.0) | 1 (0.1) | 1.00 |
| Emergency CABG, n (%) | 2 (0.2) | 0 (0.0) | 0.48 |
| GI Bleed, n (%) | 4 (0.3) | 1 (0.1) | 0.37 |
| Periprocedural MI, n (%) | 11 (0.9) | 15 (1.2) | 0.55 |
| AKI, n (%) | 4 (0.3) | 4 (0.3) | 1.00 |
| Tamponade, n (%) | 13 (1.0) | 9 (0.7) | 0.52 |
| In-hospital death, n (%) | 29 (2.3) | 16 (1.3) | 0.07 |
| In-hospital major bleed, n (%) | 32 (2.5) | 26 (2.0) | 0.51 |
| In-hospital MACCE, n (%) | 40 (3.1) | 30 (2.3) | 0.28 |
| Dissection, n (%) | 42 (3.3) | 53 (4.1) | 0.30 |
| Perforation, n (%) | 22 (1.7) | 18 (1.4) | 0.63 |
| Heartblock, n (%) | 11 (0.9) | 6 (0.5) | 0.33 |
| Slow flow, n (%) | 17 (1.3) | 10 (0.8) | 0.25 |
| Sidebranch loss, n (%) | 18 (1.4) | 14 (1.1) | 0.59 |
| Shock induction, n (%) | 12 (0.9) | 11 (0.9) | 1.00 |
| Any complication, n (%) | 99 (7.7) | 96 (7.5) | 0.88 |
| All complications, n (%) | 38 (3.0) | 45 (3.5) | 0.50 |
| Arterial haemorrhage, n (%) | 11 (0.9) | 8 (0.6) | 0.65 |
| 12-month survival, n (%) | 127 (9.9) | 107 (8.4) | 0.19 |
